# Diferença geracional no consumo alimentar de adultos brasileiros nascidos entre 1928 e 1988

**DOI:** 10.1590/0102-311XPT012225

**Published:** 2026-01-09

**Authors:** Clarisse Vasconcelos de Azevedo, Ilana Nogueira Bezerra, Thaís Meirelles de Vasconcelos

**Affiliations:** 1 Universidade Estadual do Ceará, Fortaleza, Brasil.

**Keywords:** Ingestão de Alimentos, Alimento Processado, Relação entre Gerações, Inquéritos sobre Dietas, Eating, Processed Food, Intergenerational Relations, Diet Surveys, Ingestión de Alimentos, Alimentos Procesados, Relaciones Intergeneracionales, Encuestas sobre Dietas

## Abstract

O objetivo deste estudo foi avaliar a diferença geracional no consumo alimentar de adultos brasileiros nascidos entre 1928 e 1988. Foram analisados dados do *Inquérito Nacional de Alimentação* de 2008-2009 (n = 25.324) e 2017-2018 (n = 36.480). Os alimentos consumidos foram classificados de acordo o nível de processamento, segundo a classificação NOVA. As diferenças de consumo entre gerações foram avaliadas por meio da comparação de indivíduos da mesma faixa etária em cada inquérito. Para verificar o efeito da renda sobre o consumo de ultraprocessados, foi desenvolvido um modelo de regressão linear para cada quartil de renda. As gerações mais jovens reduziram o consumo calórico total, em ambos os sexos e em todas as faixas etárias. Em 2017-2018, as gerações mais jovens, entre 20 e 39 anos, com menor nível de renda, apresentaram maior percentual de consumo de ultraprocessados em comparação às gerações mais velhas. A contribuição percentual de cada grupo da classificação NOVA para o consumo calórico total foi semelhante entre as gerações. As análises demonstraram que diferenças geracionais no consumo alimentar de adultos brasileiros nascidos entre 1928 e 1988. Entre os indivíduos de 20 a 39 anos, aqueles pertencentes às gerações mais jovens apresentaram maior consumo de ultraprocessados em comparação às gerações mais velhas, mas somente entre os grupos de menor renda.

## Abstract (en)

The aim of the present study was to assess generational differences in food consumption among Brazilian adults born between 1928 and 1988. Data from the 2008-2009 (n = 25,324) and 2017-2018 (n = 36,480) *Brazilian National Dietary Surveys* were analyzed. Foods were classified according to the degree of processing using the NOVA classification. Differences in consumption between generations were assessed by comparing individuals in the same age group in each survey. To assess the effect of income on the consumption of ultra-processed foods, a linear regression model was developed for each income quartile. A reduction in total calorie intake was found in younger generations in both sexes and all age groups. In 2017-2018, younger generations (20 to 39 years of age) with lower income levels consumed a greater frequency of ultra-processed foods compared to older generations. The percentage contribution of each NOVA classification group to total caloric intake was similar across generations. The analyses demonstrated generational differences in food consumption among Brazilian adults born between 1928 and 1988. Among individuals 20 to 39 years of age, those belonging to younger generations had a greater consumption of ultra-processed foods compared to older generations, but only among lower income groups.

## Abstract (es)

El objetivo de este estudio fue evaluar la diferencia generacional en el consumo alimentario de adultos brasileños nacidos entre 1928 y 1988. Se analizaron datos de la *Encuesta Nacional de Alimentación* de 2008-2009 (n = 25.324) y 2017-2018 (n = 36.480). Los alimentos consumidos se clasificaron según su nivel de procesamiento, de acuerdo con la clasificación NOVA. Las diferencias de consumo entre generaciones se evaluaron comparando a individuos del mismo grupo de edad en cada encuesta. Para verificar el efecto de los ingresos sobre el consumo de alimentos ultraprocesados, se desarrolló un modelo de regresión lineal para cada cuartil de ingresos. Las generaciones más jóvenes redujeron el consumo calórico total, tanto en hombres como en mujeres y en todas las franjas etarias. En 2017-2018, las generaciones más jóvenes, entre 20 y 39 años, con menor nivel de ingresos, presentaron un mayor porcentaje de consumo de alimentos ultraprocesados en comparación con las generaciones mayores. La contribución porcentual de cada grupo de la clasificación NOVA al consumo calórico total fue similar entre las generaciones. Los análisis demostraron que existen diferencias generacionales en el consumo alimentario de los adultos brasileños nacidos entre 1928 y 1988. Entre los individuos de 20 a 39 años, los pertenecientes a las generaciones más jóvenes presentaron un mayor consumo de alimentos ultraprocesados en comparación con las generaciones mayores, pero solo entre los grupos de menores ingresos.

## Introdução

Os hábitos alimentares têm passado por transformações significativas, evidenciadas, em parte, pelo aumento do consumo de alimentos ultraprocessados. Esses produtos são formulações industriais prontas para consumo, elaboradas a partir de substâncias derivadas de alimentos e acrescidas de sabores, corantes, aditivos, entre outros, com pouco ou nenhum alimento intacto em sua composição, apresentando baixa qualidade nutricional ^1^.

O consumo, bem como a disponibilidade de alimentos ultraprocessados, tem aumentado em todo o mundo, contribuindo com mais de 50% da ingestão de energia diária em países de alta renda ^2^. Achados semelhantes também vêm sendo observados em países de média e baixa renda ^3^.

Na *Pesquisa de Orçamentos Familiares* (POF) de 2017-2018 ^4^, a participação percentual no total de calorias consumidas pela população brasileira com mais de 10 anos, segundo a classificação NOVA, foi de 19,7% de alimentos ultraprocessados; 11,3% dos processados; 15,6% de ingredientes culinários e 53,4% de alimentos *in natura* ou minimamente processados. Entre 2002-2003 e 2017-2018, houve aumento do consumo de alimentos ultraprocessados nas compras de alimentos para consumo dentro do domicílio, passando de 12,6% para 18,4% do total de energia adquirida nos lares brasileiros ^4^. Já entre os períodos de 2008-2009 e 2017-2018, o consumo de *fast foods* por adultos brasileiros aumentou 17,4g/dia entre os homens e 9,5g/dia entre as mulheres ^5^.

Em paralelo a esse aumento, observa-se elevação das taxas de sobrepeso, obesidade e doenças crônicas não transmissíveis associadas à má alimentação ^6^ ^,^ ^7^. Nesse sentido, as diretrizes alimentares para a população brasileira recomendam o consumo de alimentos não processados ou minimamente processados e preparações culinárias, e a não ingestão de alimentos ultraprocessados ^8^.

Mudanças no consumo de alimentos são vivenciadas de diferentes maneiras pelos indivíduos, especialmente aqueles de diferentes gerações. As diferenças geracionais na dieta resultam da exposição compartilhada de indivíduos da mesma geração de nascimento a contextos históricos e sociais semelhantes. Essas experiências coletivas podem influenciar o estilo de vida e os comportamentos alimentares ao longo da vida, levando a hábitos divergentes entre diferentes gerações ^9^.

O momento econômico que um país atravessa e os eventos públicos marcantes criam um conjunto de acontecimentos que afetam as pessoas e definem quem elas são ^10^. Dessa forma, analisar as mudanças entre diferentes gerações de nascimento no Brasil traz novas compreensões sobre como a população brasileira alterou sua alimentação ao longo de diferentes períodos e cenários econômicos e sociais.

As estimativas de consumo de alimentos ultraprocessados no Brasil são, geralmente, apresentadas estratificadas por sexo, idade, região, área e faixas de renda. No entanto, essas análises frequentemente não consideram as diferenças geracionais em seus contextos históricos e sociais. Cada geração está associada a experiências alimentares distintas, influenciadas por fatores como a urbanização progressiva e a globalização ^11^ ^,^ ^12^.

No Brasil, apenas dois artigos investigaram as diferenças geracionais no consumo alimentar. Bezerra et al. ^13^ não avaliaram o consumo com base no nível de processamento, enquanto Costa et al. ^14^ restringiram sua pesquisa a apenas uma cidade, analisando, exclusivamente, o consumo de frutas e verduras. Sendo assim, com o objetivo de contribuir para o preenchimento dessa lacuna, este estudo buscou avaliar a diferença geracional no consumo alimentar de brasileiros nascidos entre 1928 e 1988.

## Metodologia

O estudo foi realizado com dados dos módulos de consumo alimentar das POF de 2008-2009 e 2017-2018, denominados *Inquéritos Nacionais de Alimentação* (INA) ^15^ ^,^ ^16^.

Os dois inquéritos utilizaram uma amostra mestra, formada pelo Instituto Brasileiro de Geografia e Estatística (IBGE) a partir do Sistema Integrado de Pesquisas Domiciliares, que corresponde a um conjunto de setores censitários com base nos *Censos Demográficos* de 2000 e de 2010, respectivamente. A amostragem foi definida por conglomerados, em dois estágios: no primeiro, foram sorteados os setores censitários; no segundo, os domicílios dentro de cada setor. Os setores foram submetidos à estratificação geográfica e estatística para permitir análises em diferentes domínios geográficos e socioeconômicos. Em cada POF, uma subamostra dos setores foi selecionada por amostragem aleatória simples para compor a amostra de consumo do INA ^15^ ^,^ ^16^. Detalhes sobre o processo de amostragem podem ser obtidos nas publicações oficiais no site do IBGE: https://www.ibge.gov.br/estatisticas/sociais/populacao/24786-pesquisa-de-orcamentos-familiares-2.html?=&t=microdados.

O consumo alimentar coletado na amostra do INA registrou 24,2% e 34,7% dos domicílios da amostra original, nos inquéritos de 2008-2009 e 2017-2018, respectivamente. Um total de 80.167 indivíduos com 10 anos ou mais de idade preencheram o bloco de consumo alimentar pessoal (34.003 indivíduos em 2008-2009 e 46.164 em 2017-2018). Gestantes e lactantes (n = 1.066 em 2008-2009 e n = 1.209 em 2017-2018) e indivíduos menores de 20 anos (n = 7.613 em 2008-2009 e n = 8.475 em 2017-2018) não foram considerados nas análises, totalizando 61.804 indivíduos (25.324 em 2008-2009 e 36.480 em 2017-2018).

A coleta de dados ocorreu entre maio de 2008 e abril de 2009, para a POF 2008-2009 e entre junho de 2017 e maio de 2018, para a POF 2017-2018. Os agentes de pesquisa do IBGE realizaram as entrevistas nos domicílios selecionados, por meio da aplicação de questionários. Para este trabalho, utilizou-se informações dos *Questionários de Características do Domicílio e dos Moradores* (POF 1) e do *Bloco de Consumo Alimentar Pessoal* (POF 7) ^15^ ^,^ ^16^.

Os dados socioeconômicos e as características do domicílio coletados incluem: idade, sexo (masculino e feminino), raça (branca; preta e parda; amarela e indígena), anos de estudo, área do domicílio (urbana ou rural) e renda *per capita*. A idade foi categorizada nas seguintes faixas etárias: 20-39 anos, 40-59 anos, 60-79 anos e ≥ 80 anos. Para a escolaridade, os indivíduos foram agrupados com base nos anos de estudo, considerando os ciclos de estudo no país: até 4, 5-9, 10-12 e ≥ 12. Já a renda *per capita* foi estimada pela divisão da renda total do domicílio pelo total de moradores e estratificada em quartis de renda. As gerações foram classificadas de acordo com o ano de nascimento e o período do inquérito: 2008-2009 (gerações mais velhas) e 2017-2018 (gerações mais jovens).

O consumo efetivo de alimentos no INA 2008-2009 foi coletado por meio de registros alimentares em dois dias não consecutivos no período de uma semana. Os respondentes anotaram todos os alimentos e bebidas (exceto água) consumidos ao longo de 24 horas, a quantidade consumida em medidas caseiras, o tipo de preparação, o dia da semana, o horário e o local de consumo (dentro ou fora do domicílio). Os registros foram revisados junto aos moradores por um agente de pesquisa, com base no método dos múltiplos passos ^17^. Com o intuito de facilitar o registro, os participantes também receberam uma caderneta informativa e um material instrucional com fotografias de medidas caseiras ^15^.

No INA 2017-2018, os dados do consumo alimentar foram obtidos pela aplicação de recordatórios de 24 horas (R24h) em dois dias não consecutivos, escolhidos ao longo da semana em que o agente de pesquisa permaneceu no domicílio. Os R24h foram preenchidos por um agente treinado, que entrevistou pessoalmente os indivíduos segundo o método de múltiplas passagens ^17^. Os participantes informaram todos os alimentos e bebidas (incluindo água) consumidos no dia anterior em cada uma das duas entrevistas, bem como o tipo de preparação, as porções, quantidades, os horários das refeições e o local em que elas ocorreram (dentro ou fora de casa) ^16^.

Em 2017-2018, foram realizadas perguntas de sondagem sobre a adição de azeite, manteiga, margarina, adoçante, mel, melado, maionese, *ketchup*, mostarda, molho *shoyu*, queijo ralado e creme de leite aos alimentos relatados. Para fins de comparabilidade, neste estudo, não foram considerados os alimentos de adição, uma vez que não foram investigados no inquérito de 2008-2009 ^16^. Nos dois inquéritos, foi questionado sobre o uso de açúcar e/ou adoçante para adoçar bebidas, com as opções de resposta: açúcar, adoçante, ambos ou nenhum dos dois.

As quantidades consumidas de alimentos e bebidas, reportadas em medidas caseiras, foram transformadas em gramas ou mililitros com base em uma tabela de medidas caseiras desenvolvida para o INA em cada inquérito ^15^ ^,^ ^16^. Para a conversão dos alimentos relatados em quantidades de energia e nutrientes, em ambos os inquéritos utilizou-se a versão 7.0 da Tabela Brasileira de Composição de Alimentos (TBCA) ^18^.

Em ambos os inquéritos, os itens de consumo foram classificados de acordo o nível de processamento dos alimentos, segundo Monteiro et al. ^1^, em: alimentos in natura ou minimamente processados, ingredientes culinários, alimentos processados e alimentos ultraprocessados. Em seguida, estimou-se o percentual calórico de cada grupo da classificação NOVA no consumo calórico total ^1^.

As diferenças geracionais foram avaliadas a partir da comparação de indivíduos da mesma faixa etária em cada inquérito, considerando o primeiro ano do inquérito. Por exemplo, compararam-se os indivíduos que tinham 20-39 anos em 2008 (nascidos entre 1969 e 1988) com os que tinham 20-39 anos em 2017 (nascidos entre 1978 e 1997). Essa comparação foi realizada para cada faixa etária, conforme descrito no Quadro 1.

**Quadro 1:** Descrição das gerações em relação ao ano de nascimento para cada faixa etária entre os períodos 2008-2009 e 2017-2018.

FAIXA ETÁRIA (ANOS)	PERÍODO	
	2008-2009	2017-2018
20-39	Nascidos entre 1969 e 1988	Nascidos entre 1978 e 1997
40-59	Nascidos entre 1949 e 1968	Nascidos entre 1958 e 1977
60-79	Nascidos entre 1948 e 1929	Nascidos entre 1938 e 1957
≥ 80	Nascidos até 1928	Nascidos até 1937

Para avaliar o efeito da renda sobre as diferenças geracionais no consumo de ultraprocessados, estimou-se a média do percentual de calorias provenientes desses alimentos entre os quartis de renda em cada faixa etária, comparando os nascidos em diferentes gerações.

Foram realizadas análises descritivas para caracterizar os indivíduos quanto às variáveis socioeconômicas e demográficas nos dois inquéritos. O consumo calórico e os percentuais por nível de processamento foram estimados para cada pesquisa, segundo sexo e faixa etária, juntamente com seus respectivos intervalos de 95% de confiança (IC95%). As diferenças no consumo alimentar entre as gerações foram consideradas quando não houve sobreposição dos IC95%.

Para avaliar a diferença entre as gerações, modelos de regressão linear foram desenvolvidos para cada faixa etária, tendo o ano da pesquisa (ano 1 para POF 2008-2009 e ano 2 para POF 2017-2018) como variável independente e o percentual de consumo de ultraprocessados como variável dependente. Tendo em vista que a renda é um fator diretamente relacionado ao consumo alimentar ^19^, foi desenvolvido um modelo para cada quartil de renda. Todos os modelos foram ajustados por sexo. Considerou-se a complexidade do desenho amostral e o fator de expansão da pesquisa, utilizando o programa SAS, versão online (https://www.sas.com/).

## Resultados

As características sociodemográficas da população adulta brasileira foram semelhantes nos dois inquéritos. Destaca-se aumento nos percentuais de escolaridade. Quanto à faixa etária, observa-se redução na proporção de indivíduos mais jovens (6 pontos percentuais), enquanto as faixas etárias mais avançadas aumentaram no período. Também se verificou maior percentual de indivíduos que se autodeclararam pretos e pardos no inquérito de 2017-2018. Em relação à área do domicílio, a população brasileira mostrou-se majoritariamente urbana, sendo a prevalência de indivíduos residentes nessa área aproximadamente cinco vezes maior que na área rural em ambas as pesquisas (Tabela 1).

**Tabela 1:** Características sociodemográficas da população adulta brasileira. *Pesquisas de Orçamentos Familiares*, 2008-2009 e 2017-2018.

Variáveis	2008-2009	2017-2018
% (IC95%)	% (IC95%)
Sexo		
Masculino	49,2 (48,6-49,9)	49,1 (48,5-49,7)
Feminino	50,8 (50,1-51,4)	50,9 (50,3-51,5)
Faixa etária (anos)		
20-39	46,8 (45,6-47,9)	40,6 (39,7-41,5)
40-59	35,7 (34,7-36,8)	37,3 (36,5-38,1)
60-79	15,3 (14,4-16,3)	18,9 (18,2-19,7)
≥ 80	2,2 (1,9-2,5)	3,2 (2,9-3,4)
Raça		
Branca	50,5 (49,2-51,9)	44,5 (43,4-45,6)
Preta e parda	48,3 (47,0-49,6)	54,3 (53,3-55,4)
Amarela e indígena	1,1 (0,8-1,5)	1,2 (0,9-1,4)
Escolaridade (anos)		
≤ 4	36,3 (35,1-37,5)	17,8 (17,1-18,5)
5-9	20,3 (19,4-21,2)	29,9 (29,1-30,8)
10-12	31,3 (30,2-32,5)	31,8 (31,0-32,6)
> 12	12,0 (11,2-12,9)	20,5 (19,5-21,5)
Área		
Urbana	84,4 (83,7-85,1)	86,3 (85,7-86,8)
Rural	15,6 (14,9-16,3)	13,7 (13,2-14,3)

IC95%: intervalo de 95% de confiança.

Em todas as faixas etárias, as gerações mais jovens apresentaram menor consumo calórico total em comparação às mais velhas, com diferença mais acentuada entre os indivíduos acima de 80 anos. Os homens nascidos em 1928 tiveram média de consumo calórico de 1.669kcal (IC95%: 1.509-1.829), enquanto os nascidos em 1937 apresentaram médias inferiores (1.427kcal; IC95%: 1.365-1.489). Nas mulheres, essa diferença foi de cerca de 309kcal. O menor consumo também foi observado com o aumento da idade nos dois inquéritos, independentemente do sexo e da geração de nascimento, com exceção dos indivíduos acima de 80 anos do inquérito de 2008-2009, que apresentaram consumo semelhante aos indivíduos de 60-79 anos em ambos os sexos (Tabela 2).

**Tabela 2:** Média e intervalo de 95% de confiança (IC95%) de calorias e do percentual calórico de grupos alimentares, segundo o nível de processamento, de acordo com faixa etária e geração de nascimento. *Pesquisas de Orçamentos Familiares*, 2008-2009 e 2017-2018.

	20-39 anos		40-59 anos		60-79 anos		≥ 80 anos	
	Nascidos entre 1969 e 1988	Nascidos entre 1978 e 1997	Nascidos entre 1949 e 1968	Nascidos entre 1958 e 1977	Nascidos entre 1948 e 1929	Nascidos entre 1938 e 1957	Nascidos até 1928	Nascidos até 1937
	Média (IC95%)	Média (IC95%)	Média (IC95%)	Média (IC95%)	Média (IC95%)	Média (IC95%)	Média (IC95%)	Média (IC95%)
Homens								
Calorias totais (kcal)	2.114 (2.076-2.152)	1.892 (1.860-1.924)	1.955 (1.911-1.999)	1.784 (1.754-1.815)	1.776 (1.724-1.828)	1.579 (1.541-1.617)	1.669 (1.509-1.829)	1.427 (1.365-1.489)
*In natura*/Minimamente processados (%)	56,5 (55,7-57,4)	57,8 (56,9-58,6)	59,9 (59,1-60,7)	61,3 (60,6-62,1)	64,5 (60,1-62,9)	63,1 (62,2-64,0)	64,5 (58,2-64,7)	63,3 (61,0-65,7)
Ingredientes culinários (%)	12,4 (12,1-12,7)	9,7 (9,4-10,0)	12,8 (12,5-13,1)	10,0 (9,7-10,3)	13,2 (12,6-13,7)	10,0 (9,7-10,4)	14,7 (12,7-16,8)	11,0 (9,9-12,1)
Processados (%)	12,4 (11,9-13,0)	13,2 (12,7-13,8)	13,1 (12,4-13,7)	14,3 (13,8-14,9)	12,5 (11,5-13,5)	14,9 (14,1-15,6)	11,8 (9,8-13,9)	13,0 (11,3-14,8)
Ultraprocessados (%)	18,6 (17,7-19,5)	19,3 (18,3-20,2)	14,1 (13,4-14,9)	14,3 (13,6-15,0)	12,9 (11,5-14,4)	12,0 (11,2-12,7)	12,0 (9,4-14,5)	12,6 (10,6-14,7)
Mulheres								
Calorias totais (kcal)	1.646 (1.618-1.675)	1.450 (1.426-1.475)	1.563 (1.532-1.595)	1.345 (1.325-1.366)	1.471 (1.426-1.517)	1.287 (1.261-1.313)	1.554 (1.404-1.703)	1.245 (1.198-1.291)
*In natura*/Minimamente processados (%)	54,8 (54,0-55,5)	56,9 (56,2-57,7)	58,0 (57,2-58,8)	59,5 (58,8-60,2)	60,3 (59,1-61,6)	64,5 (60,6-62,3)	59,9 (55,8-63,9)	60,5 (58,5-62,6)
Ingredientes culinários (%)	12,5 (12,2-12,9)	10,3 (10,0-10,6)	12,6 (12,3-12,8)	10,7 (10,4-11,0)	13,0 (12,5-13,5)	10,7 (10,3-11,1)	14,1 (12,8-15,5)	11,9 (10,3-13,4)
Processados (%)	12,1 (11,6-12,6)	12,0 (11,5-12,5)	12,7 (12,1-13,2)	13,5 (13,0-14,1)	12,3 (11,5-13,1)	13,0 (12,3-13,7)	11,8 (9,2-14,4)	11,9 (10,0-13,7)
Ultraprocessados (%)	20,6 (19,8-21,4)	20,8 (20,0-21,7)	16,8 (16,0-17,6)	16,3 (15,6-17,0)	14,3 (13,2-15,5)	14,8 (14,1-15,6)	14,2 (9,3-19,1)	15,7 (13,1-18,3)

Tanto em homens quanto em mulheres, a contribuição dos grupos de alimentos para o consumo calórico total foi semelhante entre as gerações, independentemente da faixa etária avaliada (Tabela 2). No entanto, a idade teve efeito importante no consumo. Entre os grupos etários mais avançados (acima de 60 anos), observaram-se diferenças na composição da dieta, com menor participação relativa de alimentos ultraprocessados e maior proporção de alimentos in natura ou minimamente processados em comparação com os indivíduos mais jovens - padrão consistente em ambos os inquéritos (Tabela 2).

**Figura 1:**
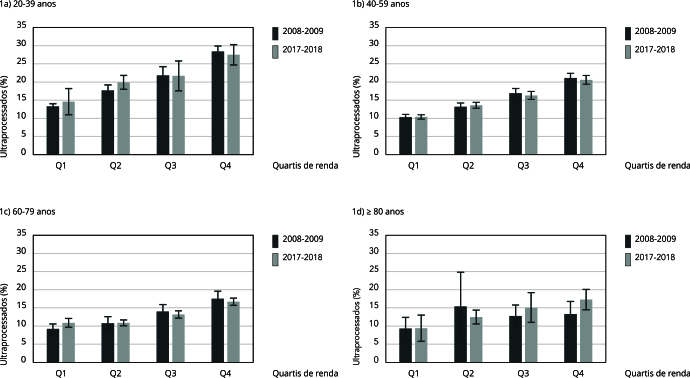
Média (ajustada por sexo) de consumo do percentual de ultraprocessados, de acordo com faixa etária, geração de nascimento e níveis de renda. *Pesquisas de Orçamentos Familiares*, 2008-2009 e 2017-2018.

A Tabela 3 mostra que o consumo de alimentos ultraprocessados aumentou entre 2008-2009 e 2017-2018, entre os adultos jovens (20-39 anos) pertencentes aos quartis de menor renda (Q1 e Q2). No segundo quartil de renda, o aumento foi mais expressivo (2,18 pontos percentuais) no mesmo período. Para as demais faixas etárias, não foi verificada associação significativa entre o consumo de alimentos ultraprocessados e os quartis renda, para o período estudado (Figura 1).

**Tabela 3:** Regressão linear entre o consumo de alimentos ultraprocessados e o ano da pesquisa, de acordo com a faixa etária e quartis de renda, ajustada por sexo. *Pesquisas de Orçamentos Familiares*, 2008-2009 e 2017-2018.

Quartis	20-39 anos		40-59 anos		60-79 anos		≥ 80 anos	
	β	Valor de p	β	Valor de p	β	Valor de p	β	Valor de p
Q1	1,32	0,0323 *	0,06	0,9119	1,72	0,0706	0,59	0,7925
Q2	2,18	0,0203 *	0,55	0,4094	0,12	0,9070	-2,71	0,5593
Q3	-0,15	0,9110	-0,61	0,4750	-0,80	0,4748	2,43	0,3533
Q4	-0,90	0,4143	-0,51	0,5969	-0,75	0,5519	4,01	0,0756

Nota: ano de referência 2008-2009.

* Significativamente diferente, p < 0,05.

## Discussão

As diferenças geracionais no consumo alimentar de 61.804 brasileiros nascidos entre 1928 e 1988 revelaram menor consumo calórico total entre as gerações mais jovens. Quando analisada a contribuição relativa dos diferentes grupos da classificação NOVA, verificou-se que a renda influenciou significativamente as diferenças no consumo de alimentos ultraprocessados. Indivíduos de 20 a 39 anos, em 2017-2018, apresentaram maior consumo de alimentos ultraprocessados em comparação com as gerações mais velhas, mas apenas entre aqueles dos menores níveis de renda.

O cenário de menor consumo calórico entre as gerações mais jovens pode ser decorrente da crise econômica ocorrida no Brasil entre 2014 e 2017, refletindo um efeito do período de coleta dos dados e não uma diferença geracional. Durante a crise, a perda do controle sobre as contas públicas reduziu a capacidade de crescimento da economia brasileira, afetando significativamente a alimentação da população e influenciando tanto o acesso quanto a qualidade dos alimentos consumidos ^20^. Nesse período, o Brasil enfrentou uma combinação de recessão econômica, aumento do desemprego, queda na renda das famílias e inflação elevada, especialmente nos preços dos alimentos ^21^, impactando diretamente a capacidade dos brasileiros de manterem uma dieta calórica adequada.

Esse quadro de crise econômica reflete-se nos indicadores de insegurança alimentar do país. Pesquisas domiciliares realizadas entre 2004 e 2018 mostram que o Brasil experimentou aumento da insegurança alimentar desde o início da crise econômica, em 2014, com forte crescimento da insegurança alimentar leve e, em menor intensidade, da moderada, de 2013 a 2017-2018 ^21^.

Por outro lado, o menor consumo calórico total, tanto em homens quanto em mulheres, pode ser reflexo da mudança no método de avaliação do consumo alimentar utilizado nas duas pesquisas. Apesar dessa limitação, em ambos os inquéritos, para reduzir divergências nas avaliações e permitir comparabilidade, a técnica de múltiplas passagens ^17^ foi aplicada para revisar os registros e coletar o R24h, com perguntas de sondagem sobre alimentos frequentemente esquecidos. Além disso, a estimativa de energia foi realizada com base na mesma tabela de composição nutricional (TBCA 7.0) ^18^.

De igual modo, estudo conduzido com 40.704 adultos da *Pesquisa de Saúde e Nutrição da China* (CHNS, acrônimo em inglês) ^22^ indicou que as gerações mais jovens apresentaram menor ingestão de energia, menor consumo de cereais e vegetais, bem como menor contribuição de carboidratos para energia, quando comparadas às gerações mais velhas.

As gerações nascidas em 1928 e 1937, classificadas como tradicionalistas ^9^, apresentaram o menor consumo calórico no presente estudo. Membros dessa geração cresceram durante a Grande Depressão e a Segunda Guerra Mundial, momentos marcados por racionamento de alimentos e escassez de recursos ^23^. Essa experiência pode ter ensinado essa geração a valorizar a economia e a evitar desperdícios, contribuído para uma maior prontidão em reduzir o consumo em períodos dedificuldades econômicas. Além disso, durante a crise de 2014 a 2017, os tradicionalistas, muitos já aposentados, podem ter enfrentado desajustes econômicos adicionais, como a perda de poder aquisitivo devido à inflação e à recessão. Em concordância, uma série temporal realizada com adultos americanos, ao examinar as diferenças por geração, revelou que indivíduos nascidos antes de 1930 experimentaram um declínio na ingestão de calorias com o aumento da idade ^24^.

Os resultados deste estudo também evidenciaram a influência da idade no consumo alimentar, indivíduos das faixas etárias mais avançadas apresentaram menor ingestão calórica e menor consumo de alimentos ultraprocessados em comparação com os mais jovens. A literatura é consistente em indicar um declínio na ingestão de energia e na quantidade de alimentos consumidos com o avanço da idade, fenômeno atribuido a mudanças fisiológicas do processo de envelhecimento, como desaceleração do metabolismo basal e perda de massa muscular, além de alterações no estilo de vida e da presença de condições médicas ^24^ ^,^ ^25^ ^,^ ^26^.

Apesar da menor ingestão calórica nas gerações mais jovens, as prevalências de obesidade e doenças crônicas têm aumentado entre adultos jovens no país ^27^ ^,^ ^28^ ^,^ ^29^. Com isso, reforça-se a hipótese de que a qualidade da alimentação tenha piorado. No entanto, os resultados deste estudo mostraram que apenas entre os indivíduos dos menores níveis de renda, o consumo de alimentos ultraprocessados foi maior nas gerações mais jovens em comparação com as mais velhas - e isso ocorreu somente entre aqueles de 20 a 39 anos.

A segunda edição do *Guia Alimentar para a População Brasileira*, publicada em 2014 ^8^, representou um marco ao recomendar explicitamente a limitação do consumo de alimentos ultraprocessados. Entretanto, sua implementação coincidiu com intenso marketing de alimentos industrializados voltado às camadas populares, o que pode explicar a maior vulnerabilidade dos jovens de baixa renda ao consumo desses produtos.

A industrialização e a globalização contribuíram para que os alimentos ultraprocessados se tornassem parte da dieta de praticamente todas as gerações ^30^ ^,^ ^31^ ^,^ ^32^. A estratégia da indústria alimentícia de desenvolver produtos destinados a diferentes faixas etárias, como alimentos prontos para consumo voltados à população idosa, pode ter contribuído para manter a participação desses produtos semelhante entre gerações ^33^.

Uma limitação dos resultados apresentados é o fato de o período de comparação das gerações ser de apenas 10 anos. Mudança nos padrões de consumo entre diferentes gerações pode levar tempo. No entanto, como ponto forte, destaca-se o ineditismo desta pesquisa, que utilizou dados representativos da população brasileira para avaliar diferenças entre gerações, podendo servir de base para futuras observações. Saber como o consumo da geração mais jovem está evoluindo dentro de um país pode auxiliar na tomada de decisões sobre importantes estratégias de saúde pública.

O indício de mudança pode ser observado nas gerações mais novas quando se comparam indivíduos nascidos entre 1969 e 1988 com os nascidos entre 1978 e 1997, denominados de geração X (1961 e 1981), e geração Y (1982 e 1996) ^9^. Essas gerações cresceram em um período de urbanização e de grande expansão da indústria de alimentos ultraprocessados, com variedade crescente de produtos industrializados ^30^. Além disso, foram expostas desde cedo a um marketing agressivo desses produtos, com propagandas voltadas especificamente para jovens e crianças, influenciando seus hábitos alimentares ^34^.

A maior contribuição calórica de alimentos ultraprocessados no consumo alimentar foi observada entre os adultos jovens, em 2017-2018 e em 2008-2009, e pode ser explicada pelo próprio desenvolvimento da cultura popular, especialmente a partir da década de 1980, com a expansão dos meios de comunicação que passaram a valorizar o consumo de alimentos ultraprocessados como parte do estilo de vida moderno ^35^. Desde então, políticas econômicas e acordos comerciais promovidos por organizações globais, com apoio de governos influentes, impulsionaram o crescimento das empresas transnacionais do setor. Essas políticas desregulamentaram a indústria, facilitaram o fluxo de capital, abriram os países ao investimento estrangeiro, permitiram que transnacionais adquirissem negócios locais e limitaram a capacidade dos governos nacionais de implementar medidas restritivas, tornando os alimentos ultraprocessados acessíveis a um número cada vez maior de pessoas ^36^.

Destaca-se também o declínio na transmissão de habilidades culinárias entre gerações, o que tem reduzido a confiança e a autonomia dos mais jovens no preparo de alimentos. Esse fenômeno, associado à desvalorização do ato de cozinhar como prática sociocultural, reflete a transição culinária em curso, reforçando a barreira ao menor consumo de alimentos in natura ou minimamente processados ^8^.

A diferença no consumo alimentar entre gerações de nascimento no Brasil foi abordada por Bezerra et al. ^13^, a partir dos dados de 15.069 adultos da linha de base da coorte multicêntrica ELSA-Brasil. A geração mais jovem (geração X) também apresentou maior consumo de produtos ultraprocessados, refrigerantes, bebidas industrializadas e carnes processadas, em relação à geração mais velha.

Um agravante dos resultados do presente estudo é o fato de ter sido encontrado maior consumo de alimentos ultraprocessados somente entre indivíduos de menor nível de renda. O nível socioeconômico tem impacto importante sobre as condições de vida, determinando o acesso a serviços, bens e produtos, entre eles os alimentos ^37^. As gerações mais jovens e de menor renda são as mais afetadas pelas mudanças econômicas. Especialmente em contextos de instabilidade, como nos períodos avaliados, essas gerações apresentam menor acumulação de riqueza e maior exposição aos desafios do mercado de trabalho. Isso também se deve à redução dos preços relativos desses produtos, à diversificação de sua oferta em diferentes locais de compras, à crescente entrada de indústrias internacionais em áreas mais remotas do país e à publicidade direcionada específicamente às comunidades de baixa renda ^36^ ^,^ ^38^.

Além disso, a influência do contexto espacial nas desigualdades socioeconômicas relacionadas à alimentação reflete a menor disponibilidade de ambientes que garantam acesso a alimentos frescos e de qualidade ^39^. As mudanças nos sistemas de abastecimento, com o surgimento de grandes redes de supermercados, contribuíram para esse cenário. Dados da POF 2008-2009 indicam que a participação de alimentos ultraprocessados era 25% maior em supermercados, onde seus preços eram 37% mais baixos em comparação com outras lojas de varejo alimentar ^40^.

Desde os anos 1990, observa-se no Brasil uma tendência de redução nos preços dos alimentos ultraprocessados, que atualmente tendem a ser mais baratos que os alimentos não processados ^41^. Tal condição elucida os limites econômicos para adesão a uma dieta baseada em alimentos frescos e nutritivos, principalmente entre grupos de baixa renda ^39^. Em concordância com os resultados desta pesquisa, o consumo de alimentos ultraprocessados aumentou significativamente nos três quintis mais baixos de renda familiar (de 13,3% para 16,8% no 1º; de 16,5% para 18,1% no 2º; e de 18% para 19,6% no 3º) entre 2008-2009 e 2017-2018 no Brasil ^42^.

Dessa forma, os achados apresentados configuram um passo importante para preencher a lacuna existente na literatura sobre diferenças geracionais no consumo alimentar no Brasil. Para todas as faixas etárias, as gerações mais jovens apresentaram menor consumo calórico total, em ambos os sexos. Além disso, a geração nascida entre 1978 e 1997 mostrou maior consumo de alimentos ultraprocessados em comparação à geração nascida entre 1969 e 1988, quando os indivíduos tinham entre 20 e 39 anos e pertenciam ao menor nível de renda. As mudanças aqui evidenciadas vão além da idade e do período em que ocorrem: demonstram alterações nos comportamentos das gerações mais jovens. que podem ter impacto importante sobre as condições de saúde da população como um todo.

## Data Availability

As fontes de informação utilizadas no estudo estão indicadas no corpo do artigo.

## References

[1] Monteiro CA, Cannon G, Levy RB, Moubarac JC, Louzada ML, Rauber F (2019). Ultra-processed foods what they are and how to identify them. Public Health Nutr.

[2] Juul F, Parekh N, Martinez-Steele E, Monteiro CA, Chang VW (2022). Ultra-processed food consumption among US adults from 2001 to 2018. Am J Clin Nutr.

[3] Dicken SJ, Qamar S, Batterham RL (2023). Who consumes ultra-processed food A systematic review of sociodemographic determinants of ultra-processed food consumption from nationally representative samples. Nutr Res Rev.

[4] Instituto Brasileiro de Geografia e Estatística (2020). Pesquisa de Orçamentos Familiares, 2017-2018: análise do consumo alimentar pessoal.

[5] Bezerra IN, Tahim JC, Rodrigues RRM, Sichieri R (2024). Changes in food consumption and prevalence of overweight and obesity in Brazilian adults between 2008 and 2018. Rev Nutr.

[6] GBD 2019 Risk Factors Collaborators (2020). Global burden of 87 risk factors in 204 countries and territories, 1990-2019: a systematic analysis for the global burden of disease study 2019.. Lancet.

[7] Dai S, Wellens J, Yang N, Li D, Wang J, Wang L (2024). Ultra-processed foods and human health: an umbrella review and updated meta-analyses of observational evidence.. Clin Nutr.

[8] Departamento de Atenção Básica. Secretaria de Atenção à Saúde. Ministério da Saúde (2014). Guia alimentar para a população brasileira.

[9] Parry E, Urwin P (2011). Generational differences in work values a review of theory and evidence. Int J Manag Rev.

[10] Howe N, Strauss W (2007). The next 20 years how customer and workforce attitudes will evolve. Harv Bus Rev.

[11] Ribeiro RM, Jesus RS (2016). A inserção da mulher no mercado de trabalho no Brasil. Revista de Ciências Humanas.

[12] Otsuka R, Yatsuya H, Tamakoshi K (2014). Descriptive epidemiologic study of food intake among Japanese adults analyses by age, time and birth cohort model. BMC Public Health.

[13] Bezerra IN, Bahamonde NMSG, Marchioni DML, Chor D, de Oliveira Cardoso L, Aquino EM (2018). Generational differences in dietary pattern among Brazilian adults born between 1934 and 1975: a latent class analysis.. Public Health Nutr.

[14] Costa GAM, Lopes MS, Lopes ACS (2022). Fruit and vegetable consumption across generations of Brazilian primary care users. Nutrition.

[15] Instituto Brasileiro de Geografia e Estatística (2011). Pesquisa de Orçamentos Familiares: 2008-2009: análise do consumo alimentar pessoal no Brasil.

[16] Instituto Brasileiro de Geografia e Estatística (2020). Pesquisa de Orçamentos Familiares 2017-2018: avaliação nutricional da disponibilidade domiciliar de alimentos no Brasil.

[17] Moshfegh AJ, Rhodes DG, Baer DJ, Murayi T, Clemens JC, Rumpler WV (2008). The US Department of Agriculture Automated Multiple-Pass Method reduces bias in the collection of energy intakes. Am J Clin Nutr.

[18] Universidade de São Paulo; Food Research Center Tabela Brasileira de Composição de Alimentos. Versão 7.1..

[19] Verly-Jr E, Oliveira DCRS, Sichieri R (2021). Custo de uma alimentação saudável e culturalmente aceitável no Brasil em 2009 e 2018. Rev Saúde Pública.

[20] Barbosa FH (2017). A crise econômica de 2014/2017. Estud Av.

[21] Jesus JG, Hoffmann R, Miranda SHG (2024). Insegurança alimentar, pobreza e distribuição de renda no Brasil. Revista de Economia e Sociologia Rural.

[22] Guo L, Huang F, Liu M, Zhang Y, Zhang J, Zhang B (2023). Generational differences in food consumption among Chinese adults of different ages. Nutrients.

[23] Wiedmer T (2015). Generations do differ best practices in leading traditionalists, boomers, and generations X, Y, and Z. Delta Kappa Gamma Bulletin.

[24] Johnston R, Poti JM, Popkin BM (2014). Eating and aging trends in dietary intake among older Americans from 1977-2010. J Nutr Health Aging.

[25] Zampelas A, Magriplis E (2020). Dietary patterns and risk of cardiovascular diseases a review of the evidence. Proc Nutr Soc.

[26] Ponti F, Santoro A, Mercatelli D, Gasperini C, Conte M, Martucci M (2020). Aging and imaging assessment of body composition: from fat to facts.. Front Endocrinol (Lausanne).

[27] Estivaleti JM, Guzman-Habinger J, Lobos J, Azeredo CM, Claro R, Ferrari G (2022). Time trends and projected obesity epidemic in Brazilian adults between 2006 and 2030. Sci Rep.

[28] Dias FSB, Silva TF, Lima YMM, Farias LS, Gadelha JG, Ramalho AA (2023). Temporal trend of severe obesity in Brazilian state capitals (2006-2021). Obesities.

[29] Garcia CAB, Meira KC, Souza AH, Oliveira ALG, Guimarães NS (2024). Obesity and associated factors in Brazilian adults systematic review and meta-analysis of representative studies. Int J Environ Res Public Health.

[30] Martins APB, Levy RB, Claro RM, Moubarac JC, Monteiro CA (2013). Increased contribution of ultra-processed food products in the Brazilian diet (1987-2009). Rev Saúde Pública.

[31] Bezerra IN, Moreira TMV, Cavalcante JB, de Moura Souza A, Sichieri R (2017). Food consumed outside the home in Brazil according to places of purchase.. Rev Saúde Pública.

[32] Downs SM, Ahmed S, Fanzo J, Herforth A (2020). Food environment typology advancing an expanded definition, framework, and methodological approach for improved characterization of wild, cultivated, and built food environments toward sustainable diets. Foods.

[33] Baker P, Machado P, Santos T, Sievert K, Backholer K, Hadjikakou M (2020). Ultra-processed foods and the nutrition transition global, regional and national trends, food systems transformations and political economy drivers. Obes Rev.

[34] Santana MO, Guimarães JS, Leite FHM, Mais LA, Horta PM, Bortoletto Martins AP (2020). Analysing persuasive marketing of ultra-processed foods on Brazilian television. Int J Public Health.

[35] Salzstein S (2006). Cultura pop astúcia e inocência. Novos Estud CEBRAP.

[36] Monteiro CA, Cannon GJ (2019). The role of the transnational ultra-processed food industry in the pandemic of obesity and its associated diseases problems and solutions. World Nutr.

[37] Antunes ABS, Cunha DB, Baltar VT, Steluti J, Pereira RA, Yokoo EM (2021). Padrões alimentares de adultos brasileiros em 2008-2009 e 2017-2018. Rev Saúde Pública.

[38] Monteiro CA, Cannon G (2012). The impact of transnational "big food" companies on the South a view from Brazil. PLoS Med.

[39] Medina LPB, Barros MBA, Sousa NFS, Bastos TF, Lima MG, Szwarcwald CL (2019). Desigualdades sociais no perfil de consumo de alimentos da população brasileira: Pesquisa Nacional de Saúde, 2013.. Rev Bras Epidemiol.

[40] Machado P, Claro R, Canella D, Sarti F, Levy R (2017). Price and convenience the influence of supermarkets on consumption of ultraprocessed foods and beverages in Brazil. Appetite.

[41] Andrade GC, Caldeira TCM, Mais LA, Bortoletto Martins AP, Claro RM (2024). Food price trends during the COVID-19 pandemic in Brazil. PLoS One.

[42] Louzada MLC, Cruz GL, Silva KAAN, Grassi AGF, Andrade GC, Rauber F (2023). Consumo de alimentos ultraprocessados no Brasil distribuição e evolução temporal 2008-2018. Rev Saúde Pública.

